# Longitudinal Data Analyses Using Linear Mixed Models in SPSS: Concepts, Procedures and Illustrations

**DOI:** 10.1100/tsw.2011.2

**Published:** 2011-01-05

**Authors:** Daniel T. L. Shek, Cecilia M. S. Ma

**Affiliations:** ^1^ Department of Applied Social Sciences, The Hong Kong Polytechnic University, Hong Kong, China; ^2^ Public Policy Research Institute, The Hong Kong Polytechnic University, Hong Kong, China; ^3^ Kiang Wu Nursing College of Macau, Macau, China; ^4^ Division of Adolescent Medicine, Department of Pediatrics, University of Kentucky College of Medicine, Lexington, KY, USA; ^5^ Department of Sociology, East China Normal University, Shanghai, China

**Keywords:** linear mixed models, hierarchical linear models, longitudinal data analysis, SPSS, Project P.A.T.H.S.

## Abstract

Although different methods are available for the analyses of longitudinal data, analyses based on generalized linear models (GLM) are criticized as violating the assumption of independence of observations. Alternatively, linear mixed models (LMM) are commonly used to understand changes in human behavior over time. In this paper, the basic concepts surrounding LMM (or hierarchical linear models) are outlined. Although SPSS is a statistical analyses package commonly used by researchers, documentation on LMM procedures in SPSS is not thorough or user friendly. With reference to this limitation, the related procedures for performing analyses based on LMM in SPSS are described. To demonstrate the application of LMM analyses in SPSS, findings based on six waves of data collected in the Project P.A.T.H.S. (Positive Adolescent Training through Holistic Social Programmes) in Hong Kong are presented.

### INTRODUCTION

How can we analyze interindividual differences in intraindividual changes over time? Traditionally, researchers used generalized linear models (GLM), such as analysis of variance (ANOVA) and analysis of covariance (ANCOVA), to examine changes in behavior across time. However, these methods would only estimate the model accurately in a balanced, repeated-measures design (e.g., equal group sizes). Unfortunately, this condition is difficult to meet and the use of the traditional univariate and multivariate test statistics might increase Type I errors under the condition of an unbalanced repeated-measures design[1,2,3].

Furthermore, the assumption of independence of observations intrinsic to GLM is not easily met when longitudinal data are under examination. As longitudinal observations may not be truly independent because of a higher-level clustering unit (i.e., time), the data used for analysis will include data that are duplicated so that observations within the clustering unit are correlated. Although it is assumed that each observation contains unique information, this information will not be truly unique, which will eventually result in biased standard errors. While violation of independence of observations is not a must in longitudinal data and there are procedures to diagnose this problem, researchers must figure out ways to deal with this problem when it exists[2,4,5].

Against the above background, there is an increased interest to study the rate of change using individual growth curve (IGC) models. IGC is an advanced technique for modeling within-person systematic change and between-person differences in developmental outcomes across different measurement waves over time. By specifying different sets of models, researchers are able to examine change in the predictive effect when additional variables are added[6]. To determine individual growth profiles and to address the questions of stability over time, researchers call for the measurement of change using this strategy[2,7]. Although the term "individual growth curve" is commonly used, it is noteworthy that analyses are usually conducted to examine "aggregates" of individual curves, rather than separate analysis of each IGC. Discussion on the use of IGC models has been described by Singer and Willett[3].

Besides capturing developmental changes over time, many researchers advocated the use of IGC when examining the longitudinal pattern of treatment effects over time[1,8,9,10] and a number of advantages of using this method were identified[1,11]. First, it does not require balanced data across different waves of data. This provides researchers with a more flexible and powerful approach when handling unbalanced data (e.g., unequal sample size, inconsistent time interval, and missing data). For example, the number and spacing of measurement occasions may vary (i.e., different points in time for different individuals), instead of being fixed (i.e., regular spaced). This is important in longitudinal studies in which the problems of participant dropout and other forms of missing measurements within individuals are often encountered. This will overcome the limitation of other conventional statistical techniques (e.g., multivariate analysis of variance [MANOVA]) that do not allow for missing data.

Second, it allows researchers to study both intra- and interindividual differences in the growth parameters (e.g., slopes and intercepts). IGC retains all of the information and variability in the data when examining the rate of changes in the dependent variables[12]. This information is valuable in the field of developmental psychology as individuals vary not only in their initial status, but also their rates of changes. Most methods for repeated-measures designs (e.g., multiple regression analyses, ANOVA, MANOVA) only focus on group differences in patterns of change, but variations of growth curve parameters might also exist at the individual level. Understanding the patterns of change and the effects at both the individual and group levels would help researchers to analyze data appropriately and capture a comprehensive picture of developmental changes across time.

Third, IGC analyses estimate the change parameters with greater precision when the number of time waves is increased. This improves the reliability of the growth parameters by reducing standard errors of the within-subject change in the growth parameters estimates[11,13]. This is obviously an advantage when compared with traditional GLM.

Fourth, the effects of predictors at higher levels (e.g., family, classroom, community, etc.) and other predictors on individual growth can flexibly be added in the growth curve models[14]. IGC can be used to explore the causal links between the linkages of predictors and changes in outcome variables across time. In addition, it allows predictors of growth to be discrete or continuous as well as time variant or time invariant. Time-variant predictors refer to independent variables that change over time (e.g., age, weight, height). Time-invariant predictors refer to independent variables that remain constant over time (e.g., gender, ethnicity).

Lastly, IGC is more powerful than other methods (e.g., ANOVA, MANOVA, multiple regression analyses) in examining the effects associated with repeated measures as it models the covariance matrix (i.e., fitting the true covariance structure to the data[15]) rather than imposing a certain type of structure as commonly used in traditional univariate and multivariate approaches[16]. In particular, the error covariance structure of the repeated measurement can be specified in IGC models, and thus allow researchers to examine true change and possible determinants of this structure during hypothesis testing. By choosing an appropriate covariance structure for the growth curve model, error variance would be reduced and allow researchers to specify a correct model that conceptualizes the patterns of change over time.

When the search term "individual growth curve" was used in September 2010, there were 260 citations in PsycINFO and 11 citations in Social Work Abstracts. When the term "growth curve modeling" was used, there were 633 and 17 citations in PsycINFO and Social Work Abstracts, respectively. These figures clearly show that there is a strong need to conduct studies on IGC modeling in the social work research context. The paucity of this analytic tool research might be related to the lack of technical papers illustrating the practical use of growth curve modeling via SPSS. There are two reasons why we document the use of linear mixed methods (LMM) in SPSS. First, SPSS is popular software used by researchers in different disciplines. As such, many researchers would like to use SPSS to perform LMM instead of using additional software. Second, there are few publications illustrating how researchers can use SPSS to analyze longitudinal data in an experimental design. As such, an illustration of how to use SPSS to analyze longitudinal intervention research would be beneficial to researchers.

The purpose of this paper is to demonstrate the use of IGC in the analyses of longitudinal data using SPSS. The general strategy for model building, testing, and comparison are described. Previous studies have illustrated the application of IGC using PROC MIXED in SAS[16,17,18], HLM[19], R[20], and SPSS[21]. Nevertheless, the longitudinal analysis reported in Peugh and Enders[21] was only a simple example not conducted within an intervention context. Furthermore, as Francis et al.[1] pointed out, "more number of time points necessitated the use of polynomial models for the individual trajectories" (p. 36). As such, the pattern of change across six time points after participating in a positive youth development program in comparison to a control group was examined in the present study. By modeling the longitudinal data, the IGC method is described and SPSS commands and outputs are examined.

### LONGITUDINAL DATA SET

The data for this study were part of a multiyear positive youth development program. Data were collected in September 2006 (Wave 1), May 2007 (Wave 2), September 2007 (Wave 3), May 2008 (Wave 4), September 2008 (Wave 5), and May 2008 (Wave 6). The majority of missing data were the result of participant absence at the day of data collection rather than attrition from the study. The number of collected questionnaires was 7,846 in Wave 1; 7,388 in Wave 2; 6,939 in Wave 3; 6,697 in Wave 4; 6,876 in Wave 5; and 6,733 in Wave 6. The number of successfully matched responses of the overall sample was 98% in Wave 1, 96% in Wave 2, 97% in Wave 3, 98% in Wave 4, 99% in Wave 5, and 97% in Wave 6. Participants that completed all six waves were 4,712 (i.e., 60% of the sample). Details are shown in Table 1.

At different measurement points, participants were required to respond to an objective outcome questionnaire, which included the Chinese Positive Youth Development Scale (CPYDS)[22]. For the purpose of illustration, a variable based on the 36 most important items (i.e., KEY 36 indicator) of the scale was focused upon (Table 2). Previous studies[22,23] showed that the CPYDS is a valid and reliable instrument.

### DATA ANALYTIC STRATEGY

The data were analyzed by using a mixed effect model with maximum likelihood (ML) estimation[24]. This method modeled individual change over time, determined the shape of the growth curves, explored systematic differences in change, and examined the effects of covariates (e.g., treatment) on group differences in the initial status and the rate of growth. It is an appropriate approach when studying individual change as it creates a two-level hierarchical model that nests time within individual[14,25].

The basic assumption of IGC is that the functional form of each individual trajectory is similar (e.g., linear growth over time in the whole sample, see Willett et al.[7]). To capture individual change over time,

**TABLE 1**
**Number of Participants at Each Measurement Occasion**

| | Wave 1 | Wave 2 | Wave 3 | Wave 4 | Wave 5 | Wave 6 |
|---|---|---|---|---|---|---|
| N (school) | 48 | 47[a] | 44[b] | 44 | 43[c] | 43 |
| No. of participants | 7,846 | 7,388 | 6,939 | 6,697 | 6,876 | 6,733 |
| Control group | 3,797 | 3,654 | 3,765 | 3,698 | 3,757 | 3,727 |
| Male | 1,936 | 1,876 | 1,896 | 1,888 | 1,874 | 1,894 |
| Female | 1,613 | 1,619 | 1,666 | 1,599 | 1,682 | 1,679 |
| Experimental group | 4,049 | 3,734 | 3,174 | 2,999 | 3,119 | 3,006 |
| Male | 2,154 | 1,998 | 1,691 | 1,548 | 1,632 | 1,591 |
| Female | 1,745 | 1,571 | 1,283 | 1,259 | 1,312 | 1,278 |

[a] One experimental school (n = 207) had withdrawn after Wave 1.

[b] Three experimental schools (n = 629) had withdrawn after Wave 2.

[c] One experimental school (n = 71) had withdrawn after Wave 4.

**TABLE 2**
**Mean KEY 36 Indicator Scores at Each Measurement Occasion**

| | Wave 1 | Wave 2 | Wave 3 | Wave 4 | Wave 5 | Wave 6 |
|---|---|---|---|---|---|---|
| Control group | 158.93 | 153.37 | 154.94 | 154.39 | 156.05 | 156.23 |
| Male | 156.81 | 153.14 | 154.60 | 154.25 | 155.94 | 156.00 |
| Female | 161.22 | 153.61 | 155.30 | 154.54 | 156.17 | 156.48 |
| Experimental group | 159.63 | 155.91 | 156.47 | 157.37 | 157.24 | 158.99 |
| Male | 156.26 | 154.92 | 154.45 | 155.78 | 154.89 | 156.96 |
| Female | 163.11 | 156.95 | 158.59 | 158.94 | 159.76 | 161.27 |

each individual trajectory is summarized by fitting to a specific form of parametric model (i.e., regressing observed record into the average of the trajectories[26]). Generally speaking, ordinary least squares (OLS) regression is used to meet this exploratory purpose[3]. Interindividual differences in growth trajectories may be found in the individual growth parameters, such as intercepts (i.e., initial status) and slopes (i.e., steep or flat). Researchers are interested in whether this heterogeneity in change is systematically related to various contextual variables[3,7]. In other words, by fitting each person's OLS trajectory to a specific parametric model, the individual trajectory in a population was obtained and allowed us to further investigate whether the individual differences in growth parameters were related to other explanatory variables.

There are two levels in IGC models. The Level 1 model refers to the within-person or intraindividual change model (i.e., repeated measurements over time). It focuses on the individual and describes the developmental changes for each individual (i.e., the variation within individual over time). The Level 1 model estimates the average within-person initial status and rate of change over time. No predictors are included in this model. The basic linear growth model is as shown below.

Level 1 model:

$$Y_{ij} = \beta_{0j} + \beta_{1j}\,(\text{Time}) + r_{ij} \qquad (1)$$

In our example, $\beta_0$ is the initial status (i.e., Wave 1) of the KEY 36 indicator for individual *i*, $\beta_1$ is the linear rate of change for individual *i*, and $r_{ij}$ is the residual in the outcome variable for individual *i* at Time *t*. $Y_{ij}$ is the repeatedly measured KEY 36 indicator for an individual *i* at Time *t*. If the effect of linear growth (*Time*, $\beta_1$) is not statistically significant, there is no need to perform further growth curve modeling analysis. To test a nonlinear individual growth trajectory across time, other higher-order polynomial trends (i.e., quadratic and cubic slopes) can also be included for model testing. This is shown in Eq. 2, in which *Time* (i.e., the linear slope, $\beta_1$) remains, while $Time^2$ (i.e., quadratic slope, $\beta_2$) and $Time^3$ (i.e., cubic slope, $\beta_3$) are added in the model.

$$Y_{ij} = \beta_{0j} + \beta_{1j}(\text{Time}) + \beta_{2j}(\text{Time}^2) + \beta_{3j}(\text{Time}^3) + r_{ij} \qquad (2)$$

The linear slope suggests that the rate of growth remains constant across time (i.e., a straight line, see Fig. 1), whereas the higher-order polynomial trends indicate that the growth rates might not be the same over time. A quadratic (second-order polynomial) individual change trajectory has no constant common slope (i.e., accelerate/decelerate over time) and consists of a single stationary point (i.e., peak/trough) (i.e., a parabola shape, see Fig. 2). A cubic trajectory has two stationary points, with one peak and one trough (i.e., S-shaped, see Fig. 3)[3].

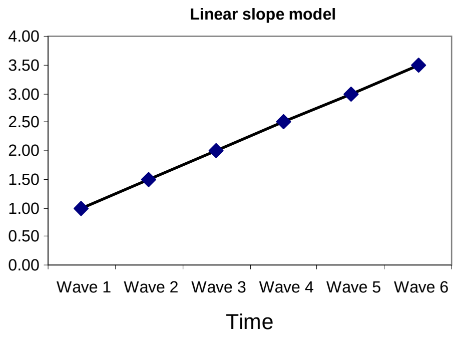

**FIGURE 1.** A hypothesized linear slope model.

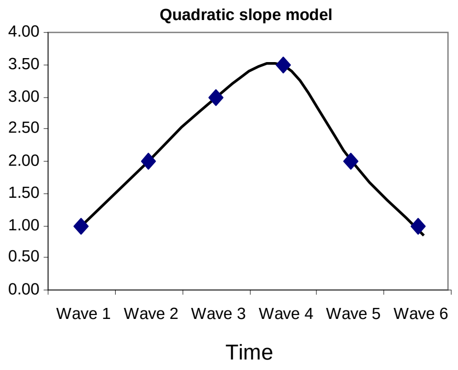

**FIGURE 2**. A hypothesized quadratic slope model.

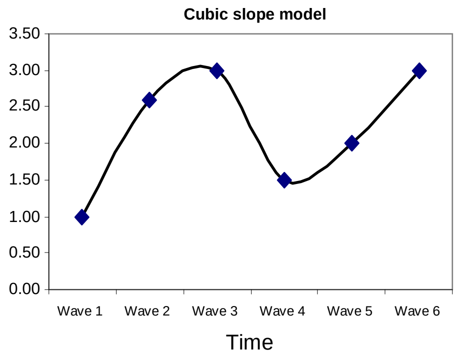

**FIGURE 3**. A hypothesized cubic slope model.

The Level 2 model captures whether the rate of change varies across individuals in a systematic way. The growth parameters (i.e., the within-subjects intercepts and slope) of Level 1 are the outcome variables to be predicted by the between-subjects variables at Level 2. At this level (Eq. 3), an explanatory variable ($W_j$) is included to analyze the predictor's effect on interindividual variation on outcome variable. The errors are assumed to be independent and normally distributed, and the variance is equal across individuals[27]. The Level 2 model is:

$$Y_{ij} = \gamma_{0i} + \gamma_{1i}(\text{Time}) + \gamma_{2i}(\text{Time}^2) + \gamma_{3i}(\text{Time}^3) + \gamma_{4i}W_j + r_{ij} \quad (3)$$

In our example, $Y_{ij}$ is the grand mean for the KEY 36 indicator for the whole sample at Time $t$. $\gamma_{0i}$ is the initial status of the KEY 36 indicator for the whole sample at Time $t$. $\gamma_{1i}$ is the linear slope of change relating to the KEY 36 indicator for the whole sample at Time $t$. $\gamma_{2i}$ is the quadratic slope of change relating to the KEY 36 indicator for the whole sample at Time $t$. $\gamma_{3i}$ is the cubic slope of change relating to the KEY 36 indicator for the whole sample at Time $t$. $\gamma_{4i}$ is used to test whether the predictor (e.g., group) is associated with the growth parameters (i.e., initial status, linear growth, quadratic growth, and cubic growth). $r_{ij}$ refers to the random effects (i.e., amount of variance) that are unexplained by the predictor.

In this study, we tested whether treatment was predictive of students' initial status and different trajectory changes in positive youth development across time. A dummy/dichotomous variable was created (i.e., *group*—control vs. experimental groups) as a predictor. Participants in the control group were coded as -1 and those in the experimental group as 1. Two covariates (i.e., gender and initial age) were included when examining the predictive program effect on the outcome variable. Gender (*k2*) was coded as -1 = male and 1 = female. A similar coding method for a dichotomous variable was found in previous studies[14,24]. For the continuous variables, a grand mean centering method was generally recommended in order to simplify the interpretation of the results[2]. In our study, the mean age was 12. *Initial age* (k1) was then centered by subtracting the mean age (i.e., age = k1 – 12) and, therefore, the centered initial age (*age*) was generated. To compute the centered initial age (*age*), the following syntax was used:

```
COMPUTE age = k1 – 12.
EXECUTE.
```

Following the strategy suggested by Singer and Willet[3], several models were tested. These included (1) an unconditional model (Model 1) that was tested to examine any mean differences in the outcome variable across individuals, (2) an unconditional growth model (Model 2) that served as a baseline model to explore whether the growth curves are linear or curvilinear, (3) two higher-order polynomial models (Models 3 and 4) that were estimated to determine if the rate of change accelerated or decelerated across time, (4) a conditional model (Model 5) that was formed to investigate whether the predictor was related to the growth parameters (i.e., initial status, linear growth, quadratic growth, and cubic growth), and (5)

three different covariance structure models (Models 6, 7, and 8) that were generated to assess the error covariance structure of the longitudinal data. The intercept and linear slope were allowed to vary across individuals. Missing data were handled through pairwise/likewise deletion.

To select the best model, -2 log likelihood (i.e., likelihood ratio test/deviance test), Akaike Information Criterion (AIC), and Bayesian Information Criterion (BIC) were used. Generally, the smaller the statistical values, the better the model fit to the data. Analyses were performed using the mixed model procedure in SPSS 17.0 statistical software[28]. In SPSS, the restricted maximum likelihood method (REML) is the default option for model estimation. As we focused on the entire model (both fixed and random effects), the maximum likelihood (ML) method was used[3]. The KEY 36 indicator (*Key*) was the outcome variable. A high score of this variable suggested better positive youth development.

### PREPARATION FOR SPSS ANALYSES

Before performing IGC analysis, a “person-period data, one record for each period” (univariate format) set is required[3]. Based on this dataset, each subject’s temporary sequenced outcome values were recoded vertically. For example, Subject ID 1234 has six records (six waves) and Subject ID 10296 has two records (two waves). The VARSTOCASES statement restructured the dataset into a “multiple-record”/stacked format[3]. The MAKE statement converted the values of a repeated-measurement variable (i.e., KEY36, SKEY, TKEY36, FKEY36, GKEY36, HKEY36) into a single variable (i.e., *key*). The INDEX statement created a new variable (i.e., *Wave*) to specify the time interval of the six measurement occasions (i.e., Wave 1 = KEY36; Wave 2 = SKEY; Wave 3 = TKEY36; Wave 4 = FKEY36; Wave 5 = GKEY36; Wave 6 = HKEY36). To convert the data into a stacked format, the following syntax was used:

```
VARSTOCASES /ID = id
/MAKE key FROM KEY36 SKEY36 TKEY36 FKEY36 GKEY36 HKEY36
/INDEX = Wave.
```

| Command | Syntax | Interpretation |
|---|---|---|
| 1 | VARSTOCASES /ID = id | Recode ID in the original file to “id” in the transposed matrix. |
| 2 | /MAKE key FROM KEY36 SKEY36 TKEY36 FKEY36 GKEY36 HKEY36 | Transform data in the six waves into one single variable. |
| 3 | /INDEX = Wave. | New variable with six values (i.e., Wave 1 to Wave 6). |

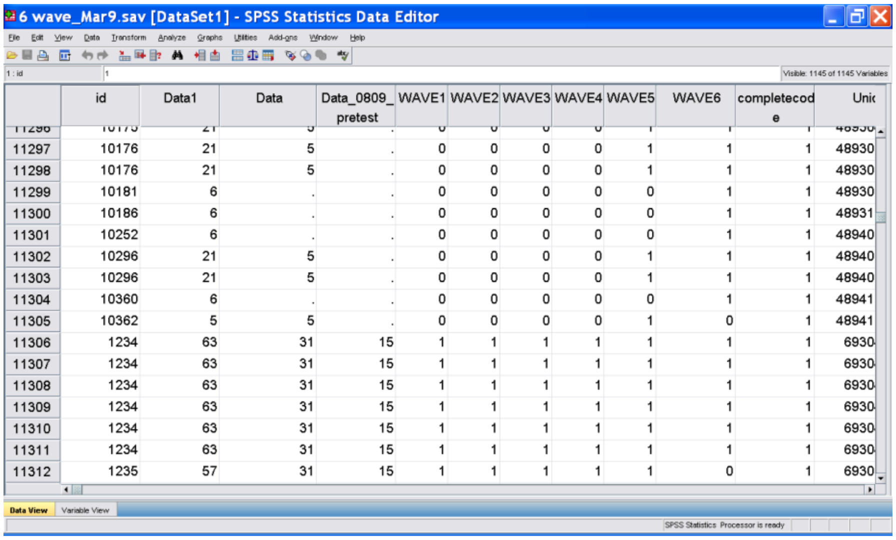

| | id | Data1 | Data | Data_0809_pretest | WAVE1 | WAVE2 | WAVE3 | WAVE4 | WAVE5 | WAVE6 | completecode | Unic |
|---|---|---|---|---|---|---|---|---|---|---|---|---|
| 11297 | 10176 | 21 | 5 | . | 0 | 0 | 0 | 0 | 1 | 1 | 1 | 48930 |
| 11298 | 10176 | 21 | 5 | . | 0 | 0 | 0 | 0 | 1 | 1 | 1 | 48930 |
| 11299 | 10181 | 6 | . | . | 0 | 0 | 0 | 0 | 0 | 1 | 1 | 48930 |
| 11300 | 10186 | 6 | . | . | 0 | 0 | 0 | 0 | 0 | 1 | 1 | 48931 |
| 11301 | 10252 | 6 | . | . | 0 | 0 | 0 | 0 | 0 | 1 | 1 | 48940 |
| 11302 | 10296 | 21 | 5 | . | 0 | 0 | 0 | 0 | 1 | 1 | 1 | 48940 |
| 11303 | 10296 | 21 | 5 | . | 0 | 0 | 0 | 0 | 1 | 1 | 1 | 48940 |
| 11304 | 10360 | 6 | . | . | 0 | 0 | 0 | 0 | 0 | 1 | 1 | 48941 |
| 11305 | 10362 | 5 | 5 | . | 0 | 0 | 0 | 0 | 1 | 0 | 1 | 48941 |
| 11306 | 1234 | 63 | 31 | 15 | 1 | 1 | 1 | 1 | 1 | 1 | 1 | 6930 |
| 11307 | 1234 | 63 | 31 | 15 | 1 | 1 | 1 | 1 | 1 | 1 | 1 | 6930 |
| 11308 | 1234 | 63 | 31 | 15 | 1 | 1 | 1 | 1 | 1 | 1 | 1 | 6930 |
| 11309 | 1234 | 63 | 31 | 15 | 1 | 1 | 1 | 1 | 1 | 1 | 1 | 6930 |
| 11310 | 1234 | 63 | 31 | 15 | 1 | 1 | 1 | 1 | 1 | 1 | 1 | 6930 |
| 11311 | 1234 | 63 | 31 | 15 | 1 | 1 | 1 | 1 | 1 | 1 | 1 | 6930 |
| 11312 | 1235 | 57 | 31 | 15 | 1 | 1 | 1 | 1 | 1 | 0 | 1 | 6930 |

One of the strengths of IGC is that it allows the irregularity of number and spacing of waves. Singer and Willett[3] highlighted the use of a time-structured predictor (TIME) for analyzing irregularly spaced datasets. In the present study, the measurement occasion was used because every individual was assessed on the same occasions. The pretest (Wave 1) values of time were set at 0, and the number of month from pretest was calculated for each wave of subsequent data collection (i.e., Waves 2–6). The data collection was scheduled at 8, 12, 20, 24, and 32 months after the baseline data collection. Therefore, *Time* was added in this model to test the linear effect of time on the KEY 36 indicator. TIME = 0 at Wave 1 (0 month, September 2006), TIME = 0.67 at Wave 2 (8 months, May 2007), TIME = 1 at Wave 3 (12 months, September 2007), TIME = 1.67 at Wave 4 (20 months, May 2008), TIME = 2 at Wave 5 (24 months, September 2008), TIME = 2.67 at Wave 6 (32 months, May 2009). By using this centering method, the dataset was organized into time structured. The relationships of the KEY 36 indicator for each measurement point are shown in Table 3.

**TABLE 3**
**Correlations of the KEY 36 Indicator across Measurement Occasions**

| | 1 | 2 | 3 | 4 | 5 | 6 |
|---|---|---|---|---|---|---|
| 1. Wave 1 | — | | | | | |
| 2. Wave 2 | 0.63** | — | | | | |
| 3. Wave 3 | 0.63** | 0.76** | — | | | |
| 4. Wave 4 | 0.57** | 0.66** | 0.71** | — | | |
| 5. Wave 5 | 0.55** | 0.64** | 0.69** | 0.77** | — | |
| 6. Wave 6 | 0.51** | 0.58** | 0.64** | 0.68** | 0.73** | — |

** $p < 0.01$.

To test a nonlinear developmental trend over the measurement period, higher-order parameters (i.e., $Time^2$, $Time^3$) were included. Six waves of data are sufficient for a precise measurement of nonlinear individual trajectories change over time[5]. The quadratic time was formed by squaring the linear term (i.e., $TIME^2$ = 0 at Wave 1; $TIME^2$ = 0.45 at Wave 2; $TIME^2$ = 1 at Wave 3; $TIME^2$ = 2.79 at Wave 4; $TIME^2$ = 4 at Wave 5; $TIME^2$ = 7.13 at Wave 6). The cubic time was also calculated by powering the linear term to three (i.e., $TIME^3$ = 0 at Wave 1; $TIME^3$ = 0.30 at Wave 2; $TIME^3$ = 1 at Wave 3; $TIME^3$ = 4.66 at Wave 4; $TIME^3$ = 8 at Wave 5; $TIME^3$ = 19.03 at Wave 6).

To compute linear function of change *Time* variable, the following syntax was used:

```
RECODE Wave (1=0) (2=.67) (3=1) (4=1.67) (5=2) (6=2.67) INTO Time.
EXECUTE.
```

The quadratic function of time (i.e., "*Time_sq*") was computed by the following syntax:

```
COMPUTE Time_sq=Time*Time.
EXECUTE.
```

The cubic function of time (i.e., "*Time_cub*") was computed by the following syntax:

```
COMPUTE Time_cub=Time*Time*Time.
EXECUTE.
```

### STEPS IN SPSS LINEAR MIXED MODEL ANALYSIS

The six waves of data from the Project P.A.T.H.S. (**P**ositive **A**dolescent **T**raining through **H**olistic **S**ocial Programmes) were used. The Project P.A.T.H.S. is a large-scale positive youth development program designed for junior secondary school students (Secondary 1 to 3, i.e., Grades 7 to 9) in Hong Kong. The details of the program are reported elsewhere[28,29,30,31].

#### Step 1: Unconditional Mean Model (Model 1)

This is a one-way ANOVA model with a random effect. In this model, no predictor is included. It serves as a baseline model to examine individual variation in the outcome variable without regard to *time*[3]. This model assesses (1) the mean of the outcome variable and (2) the amount of outcome variation that exists in intra- and interindividual levels. This latter information is important as it helps determine which level (i.e., Level 1, time variant; or Level 2, time invariant) of predictors to add when fitting the subsequent models. If the variation is high, it suggests that certain amount of outcome variation could be explained by the predictors at that level.

One of the strengths of IGC is that it examines the proportion of total outcome variation that is related to interindividual differences (i.e., intraclass correlation coefficient [ICC]). ICC describes the amount of variance in the outcome that is attributed to differences between individuals. It evaluates the necessity of modeling the nested data structure (i.e., any significant variation in individual initial status of the outcome variable). It is also a measure of the average autocorrelation of the outcome variable over time[3]. The higher value indicates the estimated average stability of the dependent variable over time.

To test the unconditional mean model, the following syntax was used:

```
mixed key
  /fixed intercept
  /random intercept | subject(id) covtype(un)
  /print solution testcov /method ml.
```

| Command | Syntax | Interpretation |
|---|---|---|
| 1 | mixed key | Requests the mixed-level analysis procedure. |
| 2 | /fixed intercept | Lists the fixed-effect variables (i.e., intercept). |
| 3 | /random intercept \| subject(id) covtype(un) | Lists the random-effect variables (i.e., intercept).<br>Specifies the classification variable (i.e., ID) and the error covariance structure type (i.e., UN). |
| 4 | /print solution testcov /method ml. | Requests the printed output with specific results (i.e., fixed-effect estimates, its standard errors, a t-test for the parameter, significance tests for the estimated variance components).<br>Specifies the use of estimation method (i.e., ML). |

The MIXED statement requests the procedure. The FIXED and RANDOM statements list the fixed and random effect variables (i.e., intercept), respectively. The SUBJECT statement specifies that ID is a classification variable to indicate that the data represent multiple observations over time for individuals. The COVTYPE statement captures the error covariance structure for data analysis. The unstructured (UN) covariance matrix for the random effects was tested (note: other covariance structure models were tested in a later section). The PRINT SOLUTION statement requested the printed output of fixed-effect estimates, its standard errors, and a t-test for the parameter. The TESTCOV is used to conduct significance tests for the estimated variance components. Maximum likelihood (ML) was used to estimate the model.

**Unconditional mean model (degrees of freedom=3)**

**Information Criteria**

| | |
|---|---|
| -2 Log Likelihood | 334969.033 |
| Akaike's Information Criterion (AIC) | 334975.033 |
| Hurvich and Tsai's Criterion (AICC) | 334975.034 |
| Bozdogan's Criterion (CAIC) | 335003.673 |
| Schwarz's Bayesian Criterion (BIC) | 335000.673 |

**Estimates of Fixed Effects**

| Parameter | Estimate | Std. Error | df | t | Sig. | 95% Confidence Interval | |
|---|---|---|---|---|---|---|---|
| | | | | | | Lower Bound | Upper Bound |
| Intercept | 155.581036 | 0.232034 | 9631.527 | 670.510 | 0.000 | 155.126201 | 156.035871 |

**Estimates of Covariance Parameters**

| Parameter | | Estimate | Std. Error | Wald Z | Sig. | 95% Confidence Interval | |
|---|---|---|---|---|---|---|---|
| | | | | | | Lower Bound | Upper Bound |
| Residual | | 228.744134 | 1.936866 | 118.100 | 0.000 | 224.979272 | 232.571997 |
| Intercept [subject = id] | Variance | 455.701266 | 7.712225 | 59.088 | 0.000 | 440.833529 | 471.070439 |

The ICC was 455.70/(455.70 + 228.74) = 0.67 (455.70/684.44), suggesting that about 67% of the total variation in the KEY 36 indicator was due to interindividual differences. In other words, the estimated average stability of the KEY36 indicator was 0.67. ICC can be used to help researchers be aware of possible mediating/moderating effects on outcome variables. If ICC is low, IGC might not perform better than the traditional method (e.g., ANOVA) in estimating fixed effects[32]. Generally, IGC is required if ICC is 0.25 or above[33,34]. The full SPSS output can be seen in Appendix A.

### Step 2: Unconditional Linear Growth Curve Model (Model 2)

This is a baseline growth curve model that examines individual variation of the growth rates (i.e., any significant variations in individual trajectory changes over time). Unlike the unconditional mean model, which only assesses the outcome variation across *individuals* (i.e., the differences between the observed mean value of each person and the true mean from the population), this model also examines *individual changes over time* (i.e., how each person’s rate of change deviates from the true rate of change of the population)[3]. If there is no interindividual difference in trajectory change over time (i.e., *Time* is not statistically significant), further model testing would not be performed.

To test the unconditional growth curve model, the following syntax was used:

```
mixed key with Time
  /fixed intercept Time
  /random intercept Time | subject(id) covtype(un)
  /print solution testcov /method ml.
```

| Command | Syntax | Interpretation |
|---|---|---|
| 1 | mixed key with Time | Requests the mixed-level analysis procedure. |
| 2. | /fixed intercept Time | Lists the fixed-effect variables (i.e., intercept, Time). |
| 3 | /random intercept Time \| subject(id) covtype(un) | Lists the random-effect variables (i.e., intercept, Time). Specifies the classification variable (i.e., ID) and the error covariance structure type (i.e., UN). |
| 4 | /print solution testcov /method ml. | Requests the printed output with specific results (i.e., fixed-effect estimates, its standard errors, a t-test for the parameter, significance tests for the estimated variance components). Specifies the use of estimation method (i.e., ML). |

In this model, WITH was specified in the MIXED statement, and *Time* was added in the MIXED and FIXED statements to test the linear growth of the KEY 36 indicator over time. Furthermore, linear slopes were allowed to randomly vary across individuals by listing *Time* in the RANDOM statement.

**Unconditional linear growth model (degrees of freedom=6)**

**Information Criteria**

| | |
|---|---|
| -2 Log Likelihood | 334025.838 |
| Akaike's Information Criterion (AIC) | 334037.838 |
| Hurvich and Tsai's Criterion (AICC) | 334037.841 |
| Bozdogan's Criterion (CAIC) | 334095.119 |
| Schwarz's Bayesian Criterion (BIC) | 334089.119 |

**Estimates of Fixed Effects**

| Parameter | Estimate | Std. Error | df | t | Sig. | 95% Confidence Interval Lower Bound | 95% Confidence Interval Upper Bound |
|---|---|---|---|---|---|---|---|
| Intercept | 155.910860 | 0.274810 | 8822.914 | 567.340 | 0.000 | 155.372167 | 156.449552 |
| Time | -0.328023 | 0.116614 | 6778.905 | -2.813 | 0.005 | -0.556623 | -0.099423 |

**Estimates of Covariance Parameters**

| Parameter | | Estimate | Std. Error | Wald Z | Sig. | 95% Confidence Interval Lower Bound | 95% Confidence Interval Upper Bound |
|---|---|---|---|---|---|---|---|
| Residual | | 192.113758 | 1.889325 | 101.684 | 0.000 | 188.446209 | 195.852685 |
| Intercept + Time [subject = id] | UN (1,1) | 532.888716 | 10.727355 | 49.676 | 0.000 | 512.272862 | 554.334232 |
| | UN (2,1) | -55.623597 | 3.644517 | -15.262 | 0.000 | -62.766718 | -48.480475 |
| | UN (2,2) | 42.537662 | 1.830458 | 23.239 | 0.000 | 39.097156 | 46.280929 |

The significant values in both the intercept and linear slope parameters indicate that the initial status and linear growth rate were not constant over time. There was a significant linear decrease in the KEY 36 indicator scores ($\beta = -0.33$, $SE = 0.12$, $p < 0.01$). The mean estimated initial status and linear growth rate for the sample were 155.91 and -0.33, respectively. This suggested that the mean KEY 36 indicator was 155.91 and decreased with time. The random error terms associated with the intercept and linear effect were significant ($p < 0.01$), suggesting that the variability in these parameters could be explained by between-individual predictors.

Comparing within-individual variation in initial status between Model 1 and Model 2, there was a decline in the residual variance of 36.63 (228.74 to 192.11). This suggested that about 37% of the within-individual variation in the KEY 36 indicator was associated with linear rate of change.

The correlation (β = -55.62, *SE* = 3.64, $p < 0.01$) between the intercept and the linear growth parameter was negative. This suggests that students with high KEY 36 indicator scores had a slower linear decrease, whereas students with low KEY 36 indicator scores had a faster decrease in linear growth over time.

### Step 3: Quadratic Growth Curve Model (Higher-Order Change Trajectories) (Model 3)

Individual growth trajectories are usually nonlinear over time as shown in previous developmental studies[35,36]. As such, two higher-order polynomial models were tested. The analyses examined whether the rate of growth accelerated or decelerated over time. To test the quadratic rate of change, a model with quadratic time (*Time_sq*) was examined by adding quadratic parameter in the previous model.

To test the quadratic effect, the following syntax was used:

```
mixed key with Time Time_sq
  /fixed intercept Time Time_sq
  /random intercept time | subject(id) covtype(un)
  /print solution testcov /method ml.
```

| Command | Syntax | Interpretation |
|---|---|---|
| 1 | mixed key with Time Time_sq | Requests the mixed-level analysis procedure. |
| 2. | /fixed intercept Time Time_sq | Lists the fixed-effect variables (i.e., intercept, Time, Time_sq). |
| 3 | /random intercept Time \| subject(id) covtype(un) | Lists the random-effect variables (i.e., intercept, Time).<br>Specifies the classification variable (i.e., ID) and the error covariance structure type (i.e., UN). |
| 4 | /print solution testcov /method ml. | Requests the printed output with specific results (i.e., fixed-effect estimates, its standard errors, a t-test for the parameter, significance tests for the estimated variance components).<br>Specifies the use of estimation method (i.e., ML). |

#### Quadratic growth model (degrees of freedom=7)

**Information Criteria**

| | |
|---|---|
| -2 Log Likelihood | 333749.959 |
| Akaike's Information Criterion (AIC) | 333763.959 |
| Hurvich and Tsai's Criterion (AICC) | 333763.962 |
| Bozdogan's Criterion (CAIC) | 333830.786 |
| Schwarz's Bayesian Criterion (BIC) | 333823.786 |

**Estimates of Fixed Effects**

| Parameter | Estimate | Std. Error | df | t | Sig. | 95% Confidence Interval | |
|---|---|---|---|---|---|---|---|
| | | | | | | Lower Bound | Upper Bound |
| Intercept | 157.685999 | 0.294702 | 11121.113 | 535.069 | 0.000 | 157.108331 | 158.263667 |
| Time | -5.048185 | 0.306463 | 29742.869 | -16.472 | 0.000 | -5.648866 | -4.447505 |
| Time_sq | 1.747592 | 0.104963 | 25301.492 | 16.650 | 0.000 | 1.541859 | 1.953325 |

**Estimates of Covariance Parameters**

| Parameter | | Estimate | Std. Error | Wald Z | Sig. | 95% Confidence Interval | |
|---|---|---|---|---|---|---|---|
| | | | | | | Lower Bound | Upper Bound |
| Residual | | 190.085974 | 1.867786 | 101.771 | 0.000 | 186.460206 | 193.782246 |
| Intercept + Time [subject = id] | UN (1,1) | 533.975708 | 10.706157 | 49.876 | 0.000 | 513.398977 | 555.377142 |
| | UN (2,1) | -55.958842 | 3.626588 | -15.430 | 0.000 | -63.066823 | -48.850860 |
| | UN (2,2) | 42.535485 | 1.815904 | 23.424 | 0.000 | 39.121213 | 46.247735 |

Results showed that all growth parameters were significant ($p$ < 0.01), indicating that there were significant between-subjects variations in the initial status, and linear and quadratic time trajectories (i.e., reliably different from zero). The initial status (grand mean score at Wave 1) of the KEY 36 indicator was 157.69 (β = 157.69, *SE* = 0.29, $p$ < 0.01). The significant linear effect for the KEY 36 indicator was negative (β = -5.05, *SE* = 0.31, $p$ < 0.01), revealing that the rate of linear growth decreased over time. The significant quadratic effect was positive (β = 1.75, *SE* = 0.10, $p$ < 0.01), showing that the rate of growth increased over time. The expected deceleration was found after Wave 1 [-5.05 / (2 (1.75)) = 1.44][3]. This indicates that the decreasing effect gradually diminished after Wave 1 (i.e., U-shaped curve). Compared to the linear change trajectory (-5.05), the rate of quadratic growth (1.75) was small. Based on the above results, it showed that the KEY 36 indicator decreased at the beginning, but this trend slowed down later on.

Given that the quadratic model improved model fit over the linear model ($\chi^2$ (1) = 334025.84 – 333749.96 = 275.88, $p$ < 0.01; Δ AIC = 334037.84 – 333763.96 = 273.88, $p$ < 0.01; Δ BIC = 334089.12 – 333823.79 = 265.33), both linear and quadratic growth curve parameters were retained in the subsequent models. It indicated that the potential of curvature trajectories fit the data better.

### Step 4: Cubic Growth Curve Model (Higher-Order Change Trajectories) (Model 4)

Researchers noted that more number of time points was required when testing different types of polynomial models for the individual trajectories, even though it is difficult to interpret such a complex model[3]. A cubic model was also tested to summarize individual change for the whole sample. The syntax of this model was similar to the previous one, except with the inclusion of *Time_cub* in the FIXED statement. The purpose of this model is to test any cubic changes in individual trajectories over time (i.e., examine whether another nonlinear growth model fits the data better).

The following syntax was used:

```
mixed key with Time Time_sq Time_cub
  /fixed intercept Time Time_sq Time_cub
  /random intercept time | subject(id) covtype(un)
  /print solution testcov /method ml.
```

| Command | Syntax | Interpretation |
|---|---|---|
| 1 | mixed key with Time Time_sq Time_cub | Requests the mixed-level analysis procedure. |
| 2. | /fixed intercept Time Time_sq Time_cub | Lists the fixed-effect variables (i.e., intercept, Time, Time_sq, Time_cub). |
| 3 | /random intercept Time \| subject(id) covtype(un) | Lists the random-effect variables (i.e., intercept, Time).<br>Specifies the classification variable (i.e., ID) and the covariance structure type (i.e., UN). |
| 4 | /print solution testcov /method ml. | Requests the printed output with specific results (i.e., fixed-effect estimates, its standard errors, a t-test for the parameter, significance tests for the estimated variance components).<br>Specifies the use of estimation method (i.e., ML). |

### Cubic growth model (degrees of freedom=8)

**Information Criteria**

| | |
|---|---|
| -2 Log Likelihood | 333684.554 |
| Akaike's Information Criterion (AIC) | 333700.554 |
| Hurvich and Tsai's Criterion (AICC) | 333700.558 |
| Bozdogan's Criterion (CAIC) | 333776.928 |
| Schwarz's Bayesian Criterion (BIC) | 333768.928 |

**Estimates of Fixed Effects**

| | | | | | | 95% Confidence Interval | |
|---|---|---|---|---|---|---|---|
| Parameter | Estimate | Std. Error | df | t | Sig. | Lower Bound | Upper Bound |
| Intercept | 158.200143 | 0.301451 | 12002.213 | 524.796 | 0.000 | 157.609251 | 158.791035 |
| Time | -9.459394 | 0.625204 | 25657.553 | -15.130 | 0.000 | -10.684829 | -8.233958 |
| Time_sq | 6.261327 | 0.567495 | 23457.836 | 11.033 | 0.000 | 5.148999 | 7.373655 |
| Time_cub | -1.122656 | 0.138710 | 23043.425 | -8.094 | 0.000 | -1.394538 | -0.850774 |

**Estimates of Covariance Parameters**

| | | | | | | 95% Confidence Interval | |
|---|---|---|---|---|---|---|---|
| Parameter | | Estimate | Std. Error | Wald Z | Sig. | Lower Bound | Upper Bound |
| Residual | | 189.500331 | 1.862289 | 101.757 | 0.000 | 185.885238 | 193.185729 |
| Intercept + Time [subject = id] | UN (1,1) | 534.401214 | 10.704881 | 49.921 | 0.000 | 513.826567 | 555.799710 |
| | UN (2,1) | -56.258192 | 3.627004 | -15.511 | 0.000 | -63.366989 | -49.149395 |
| | UN (2,2) | 42.749224 | 1.817371 | 23.523 | 0.000 | 39.331602 | 46.463812 |

*Time*, *Time_sq*, and *Time_cub* had a significant contribution in the model ($p$ < 0.01). The negative effect of linear growth (β = -9.46, *SE* = 0.63, $p$ < 0.01) suggested that the KEY 36 indicator decreased at the beginning. The positive effect of quadratic growth (β = 6.26, *SE* = 0.57, $p$ < 0.01) indicated a deceleration in the rate of change (i.e., initially decreased and then began to increase). However, the negative effect of cubic growth (β = -1.12, *SE* = 0.14, $p$ < 0.01) revealed that such deceleration gradually diminished over time. Given that the cubic model improved model fit over the previous model ($\chi^2$ (1) = 333749.96 – 333684.55 = 65.41, $p$ < 0.01; Δ AIC = 333763.96 – 333700.55 = 63.41, $p$ < 0.01; Δ BIC = 333823.79 – 33768.93 = 54.86), cubic growth curve parameters were retained in the subsequent models.

### Step 5: Adding Predictors (Model 5)

To test the predictor effect on the shape of individual growth trajectories, a dichotomous variable "*group*" was examined as a time-invariant covariate to explore any group differences in change over time (i.e., interaction with time). It examined whether *group* was a predictor of the intercept, linear, quadratic, and cubic parameters. In particular, the relationships between the KEY 36 indicator and group were estimated after controlling the effect of gender "*k2*" and initial age "*age*".

The following syntax was used:

```
mixed key with Time Time_sq  Time_cub group  k2 age
  /fixed Time Time_sq Time_cub group k2 age group*Time group*Time_sq group*Time_cub k2*Time
k2*Time_sq k2*Time_cub age*Time age*Time_sq  age*Time_cub
  /random intercept time | subject(id) covtype(un)
  /print solution testcov /method ml.
```

| Command | Syntax | Interpretation |
|---|---|---|
| 1 | mixed key with Time Time_sq Time_cub k2 age | Requests the mixed-level analysis procedure. |
| 2. | /fixed intercept Time Time_sq Time_cub group k2 age group*Time group*Time_sq group*Time_cub k2*Time k2*Time_sq k2*Time_cub age*Time age*Time_sq age*Time_cub | Lists the fixed-effect variables (i.e., intercept, Time Time_sq Time_cub group k2 age group*Time group*Time_sq group*Time_cub k2*Time k2*Time_sq k2*Time_cub age*Time age*Time_sq age*Time_cub). |
| 3 | /random intercept Time \| subject(id) covtype(un) | Lists the random-effect variables (i.e., intercept, Time).<br>Specifies the classification variable (i.e., ID) and the error covariance structure type (i.e., UN). |
| 4 | /print solution testcov /method ml. | Requests the printed output with specific results (i.e., fixed-effect estimates, its standard errors, a t-test for the parameter, significance tests for the estimated variance components).<br>Specifies the use of estimation method (i.e., ML). |

**Information Criteria**

| | |
|---|---|
| -2 Log Likelihood | 233789.704 |
| Akaike's Information Criterion (AIC) | 233829.704 |
| Hurvich and Tsai's Criterion (AICC) | 233829.735 |
| Bozdogan's Criterion (CAIC) | 234013.718 |
| Schwarz's Bayesian Criterion (BIC) | 233993.718 |

**Estimates of Fixed Effects**

| Parameter | Estimate | Std. Error | df | t | Sig. | 95% Confidence Interval Lower Bound | 95% Confidence Interval Upper Bound |
|---|---|---|---|---|---|---|---|
| Intercept | 156.092067 | 0.499113 | 7690.645 | 312.739 | 0.000 | 155.113669 | 157.070465 |
| Time | -5.622431 | 0.997006 | 18597.867 | -5.639 | 0.000 | -7.576654 | -3.668209 |
| Time_sq | 4.076358 | 0.920552 | 17140.516 | 4.428 | 0.000 | 2.271981 | 5.880735 |
| Time_cub | -0.760958 | 0.227309 | 16900.036 | -3.348 | 0.001 | -1.206507 | -0.315409 |
| group | 0.057759 | 0.357038 | 7489.439 | 0.162 | 0.871 | -0.642136 | 0.757654 |
| k2 | 5.961435 | 0.703101 | 7462.870 | 8.479 | 0.000 | 4.583158 | 7.339711 |
| age | -1.686331 | 0.391491 | 7413.377 | -4.307 | 0.000 | -2.453764 | -0.918898 |
| group * Time | 2.921715 | 0.701601 | 18243.057 | 4.164 | 0.000 | 1.546512 | 4.296918 |
| group * Time_sq | -2.381432 | 0.652016 | 16943.156 | -3.652 | 0.000 | -3.659452 | -1.103412 |
| group * Time_cub | 0.534979 | 0.161594 | 16751.241 | 3.311 | 0.001 | 0.218238 | 0.851721 |
| k2 * Time | -9.876641 | 1.380947 | 18282.678 | -7.152 | 0.000 | -12.583428 | -7.169855 |
| k2 * Time_sq | 6.117306 | 1.278788 | 16907.613 | 4.784 | 0.000 | 3.610749 | 8.623864 |
| k2 * Time_cub | -1.156036 | 0.316100 | 16727.092 | -3.657 | 0.000 | -1.775624 | -0.536447 |
| age * Time | 3.191449 | 0.805365 | 18480.635 | 3.963 | 0.000 | 1.612860 | 4.770038 |
| age * Time_sq | -2.199849 | 0.755895 | 17145.136 | -2.910 | 0.004 | -3.681479 | -0.718218 |
| age * Time_cub | 0.449315 | 0.187762 | 16915.750 | 2.393 | 0.017 | 0.081281 | 0.817348 |

**Estimates of Covariance Parameters**

| Parameter | | Estimate | Std. Error | Wald Z | Sig. | 95% Confidence Interval Lower Bound | 95% Confidence Interval Upper Bound |
|---|---|---|---|---|---|---|---|
| Residual | | 179.357492 | 2.026818 | 88.492 | 0.000 | 175.428671 | 183.374300 |
| Intercept + Time [subject = id] | UN (1,1) | 502.629872 | 11.869756 | 42.345 | 0.000 | 479.895762 | 526.440966 |
| | UN (2,1) | -48.229541 | 3.859513 | -12.496 | 0.000 | -55.794049 | -40.665034 |
| | UN (2,2) | 41.665972 | 1.952382 | 21.341 | 0.000 | 38.009834 | 45.673792 |

*Group* was a significant predictor of the linear, quadratic, and cubic changes in the KEY36 indicator ($p < 0.01$), but not associated with the initial status (β = 0.06, SE = 0.36, $p < 0.01$). Regarding the linear slope of the KEY 36 indicator, the control group showed a faster rate of change as compared with the experimental group (β = 2.92, $t = 4.16$, $p < 0.01$). In terms of quadratic growth, the control group had a slower rate of change in the KEY 36 indicator when compared with the experimental group (β = -2.38, $t = -3.65$, $p < 0.01$). Lastly, the control group had a faster rate of cubic change than the experimental group (β = 0.53, $t = 3.31$, $p < 0.01$). In other words, a stable trajectory of the KEY 36 indicator was found in the experimental group, but not in the control group. The predictor (*group*) accounted for 7% [(192.11 – 179.36) / 192.11 = 0.066] of the within-individual variations in the KEY 36 indicator. This shows that only 7% of the overall variability in the KEY36 indicator is explained by *Group*.

Singer and Willett[3] proposed using prototypical values to demonstrate the effect of treatment on initial status and the rate of change across time. The step in creating prototypical plots is generally identical to the method of plotting graphs in regression[37]. We can obtain the fitted trajectories by substituting the two values of *Group* in the cubic model: $Y_{ij} = 156.09 + (-5.62)(\text{Time}) + (4.08)(\text{Time}^2) + (-0.76)(\text{Time}^3) + (0.06)(\text{Group}) + (2.92)(\text{Group X Time}) + (-2.38)(\text{Group X Time}^2) + (0.53)(\text{Group X Time}^3)$. This method was also used in previous studies[38,39]. The trajectories of the control and experimental groups are shown in Fig. 4. In general, the experimental group had a steady growth rate of change in the KEY 36 indicator compared to the control group.

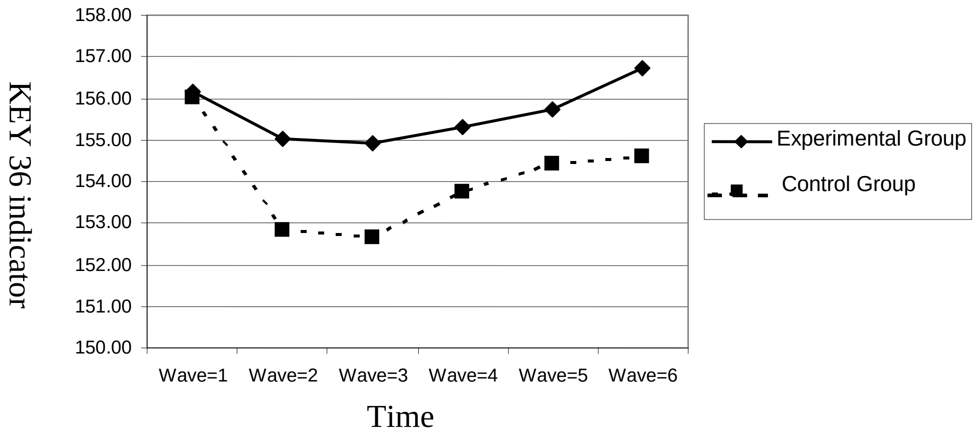

**FIGURE 4**. Fitted trajectories of the control and experimental groups.

*For control group (-1),*

$Y_{ij}$ = 156.09 + (-5.62)(Time) + (4.08)($Time^2$) + (-0.76)($Time^3$) + (0.06)(-1) + (2.92)(-1)(Time) + (-2.38)(-1)($Time^2$) + (0.53)(-1)($Time^3$)

$Y_{ij}$ = 156.03 – 8.54(Time) + 6.46($Time^2$) – 1.29($Time^3$)

*For experimental group (1),*

$Y_{ij}$ = 156.09 + (-5.62)(Time) + (4.08)($Time^2$) + (-0.76)($Time^3$) + (0.06)(1) + (2.92)(1)(Time) + (-2.38)(1)($Time^2$) + (0.53)(1)($Time^3$)

$Y_{ij}$ = 156.15 – 2.70(Time) + 1.7($Time^2$) – 0.23($Time^3$)

### Step 6: Examining Covariance Structure

One of the advantages of IGC is the availability to specify the within-individual error covariance structure that best fits the data. The purpose of testing different error covariance matrices is to describe how the error is distributed[18]. It examines whether the properties imposed on the error covariance structure of the parametric model fit well to the data[3]. This is very important when we examine unequally spaced and unbalanced data, which are commonly found in longitudinal studies.

In fact, studies showed that the estimated variances of the parameter estimates are likely to be biased and inconsistent when repeated measurements are taken on the same individual across time (i.e., failure to take account for heteroscedasticity)[40,41] and consequently affect the precision of estimating the appropriate model[42]. Researchers advocated the use of this variance-covariance testing approach as it improves model predictions and statistic inferences, especially when examining random effects models[43,44]. In the present study, three types of covariance structures (i.e., unstructured, compound symmetric, and first-order autoregressive) that were commonly examined in previous studies were tested[18,45,46,47].

#### *Unstructured (UN) Covariance Structure (Model 6)*

The unstructured covariance structure model often offers the best fit and is most commonly found in longitudinal data as it is the most parsimonious, which requires no assumption in the error structure[18]. In this model, the syntax is the same as the previous one, except for the inclusion of the REPEATED statement, which substitutes the RANDOM statement. The REPEATED statement lists “wave”, which specified the number of each repeated measurement (e.g., 1, 2, 3….). The SUBJECT statement identified the unit of analysis on which repeated observations were measured. The COVTYPE specified an unstructured (UN) residual covariance structure in which the variance between waves is not constant and the correlations between waves are differed across time.

The following syntax was used:

```
mixed key with Time Time_sq Time_cub group  k2 age
  /fixed Time Time_sq Time_cub group k2 age group*Time group*Time_sq group*Time_cub k2*Time
k2*Time_sq k2*Time_cub age*Time age*Time_sq  age*Time_cub
  /repeated wave | subject(id) covtype(un)
  /print solution testcov /method ml.
```

| Command | Syntax | Interpretation |
|---|---|---|
| 1 | mixed key with Time Time_sq Time_cub k2 age | Requests the mixed-level analysis procedure. |
| 2. | /fixed intercept Time Time_sq Time_cub group k2 age group*Time group*Time_sq group*Time_cub k2*Time k2*Time_sq k2*Time_cub age*Time age*Time_sq age*Time_cub | Lists the fixed-effect variables (i.e., intercept, Time Time_sq Time_cub group k2 age group*Time group*Time_sq group*Time_cub k2*Time k2*Time_sq k2*Time_cub age*Time age*Time_sq age*Time_cub). |
| 3 | /repeated wave \| subject(id) covtype(un) | Specifies the error covariance structure type (i.e., UN). |
| 4 | /print solution testcov /method ml. | Requests the printed output with specific results (i.e., fixed-effect estimates, its standard errors, a t-test for the parameter, significance tests for the estimated variance components). |
| | | Specifies the use of estimation method (i.e., ML). |

**Information Criteria**

| | |
|---|---|
| -2 Log Likelihood | 233203.108 |
| Akaike's Information Criterion (AIC) | 233277.108 |
| Hurvich and Tsai's Criterion (AICC) | 233277.213 |
| Bozdogan's Criterion (CAIC) | 233617.534 |
| Schwarz's Bayesian Criterion (BIC) | 233580.534 |

### *Compound Symmetry (CS) Covariance Structure (Model 7)*

To examine whether the variance and correlation between each pair of observations are constant across time points, a compound symmetry (CS) covariance structure was tested. Therefore, the specification of CS was added in the COVTYPE statement.

The following syntax was used:

```
mixed key with Time Time_sq Time_cub group k2 age
  /fixed Time Time_sq Time_cub group k2 age group*Time group*Time_sq group*Time_cub k2*Time
k2*Time_sq k2*Time_cub age*Time age*Time_sq age*Time_cub
  /repeated wave | subject(id) covtype(CS)
  /print solution testcov /method ml.
```

| Command | Syntax | Interpretation |
|---|---|---|
| 1 | mixed key with Time Time_sq Time_cub k2 age | Requests the mixed-level analysis procedure. |
| 2. | /fixed intercept Time Time_sq Time_cub group k2 age group*Time group*Time_sq group*Time_cub k2*Time k2*Time_sq k2*Time_cub age*Time age*Time_sq age*Time_cub | Lists the fixed-effect variables (i.e., intercept, Time Time_sq Time_cub group k2 age group*Time group*Time_sq group*Time_cub k2*Time k2*Time_sq k2*Time_cub age*Time age*Time_sq age*Time_cub). |
| 3 | /repeated wave \| subject(id) covtype(cs) | Specifies the error covariance structure type (i.e., CS). |
| 4 | /print solution testcov /method ml. | Requests the printed output with specific results (i.e., fixed-effect estimates, its standard errors, a t-test for the parameter, significance tests for the estimated variance components).<br>Specifies the use of estimation method (i.e., ML). |

**Information Criteria**

| | |
|---|---|
| -2 Log Likelihood | 234605.557 |
| Akaike's Information Criterion (AIC) | 234641.557 |
| Hurvich and Tsai's Criterion (AICC) | 234641.583 |
| Bozdogan's Criterion (CAIC) | 234807.170 |
| Schwarz's Bayesian Criterion (BIC) | 234789.170 |

### *First-Order Autoregressive (AR1) Covariance Structure (Model 8)*

In this model, the variance is assumed to be heterogeneous and the correlations between the two adjacent time points decline across measurement occasions. The AR1 was specified in the COVTYPE statement.

The following syntax was used:

```
mixed key with Time Time_sq Time_cub group k2 age
  /fixed Time Time_sq Time_cub group k2 age group*Time group*Time_sq group*Time_cub k2*Time
k2*Time_sq k2*Time_cub age*Time age*Time_sq age*Time_cub
  /repeated wave | subject(id) covtype(AR1)
  /print solution testcov /method ml.
```

| Command | Syntax | Interpretation |
|---|---|---|
| 1 | mixed key with Time Time_sq Time_cub k2 age | Requests the mixed-level analysis procedure. |
| 2. | /fixed intercept Time Time_sq Time_cub group k2 age group*Time group*Time_sq group*Time_cub k2*Time k2*Time_sq k2*Time_cub age*Time age*Time_sq age*Time_cub | Lists the fixed-effect variables (i.e., intercept, Time Time_sq Time_cub group k2 age group*Time group*Time_sq group*Time_cub k2*Time k2*Time_sq k2*Time_cub age*Time age*Time_sq age*Time_cub). |
| 3 | /repeated wave \| subject(id) covtype(AR1) | Specifies the error covariance structure type (i.e., AR1). |
| 4 | /print solution testcov /method ml. | Requests the printed output with specific results (i.e., fixed-effect estimates, its standard errors, a t-test for the parameter, significance tests for the estimated variance components).<br>Specifies the use of estimation method (i.e., ML). |

**Information Criteria**

| | |
|---|---|
| -2 Log Likelihood | 234964.276 |
| Akaike's Information Criterion (AIC) | 235000.276 |
| Hurvich and Tsai's Criterion (AICC) | 235000.301 |
| Bozdogan's Criterion (CAIC) | 235165.888 |
| Schwarz's Bayesian Criterion (BIC) | 235147.888 |

Based on Table 4, it was observed that the smallest values in the three fit criterion (i.e., -2 times the log-likelihood [-2LL], Akaike's Information Criteriaon [AIC], and the Bayesian Information Criterion [BIC]) were found in the UN model. These differences were statistically significant when compared to the results of a chi-square distribution with degrees of freedom (i.e., 37 parameters-18 parameters, $\Delta df = 19$, $p < 0.01$). This suggested that the UN model was the best model in fitting the data, although it required many parameters. The correlated error terms and heterogeneous variances might be the result of unequally spaced time points of measurement. If the time points were closely spaced, the possibility of modeling correlated errors might be higher than those that were scheduled far apart[2]. The use of this variance-covariance approach would improve model predictions. Readers interested in different error covariance structures can read the work of others for details[47,48,49,50].

**TABLE 4**
**Results of Information Criterion among Three Covariance Structure Models**

| Covariance Structure | -2LL | AIC | BIC |
|---|---|---|---|
| Unstructured (df=37) | 233203.11 | 233277.11 | 233580.53 |
| Compound symmetry (df=18) | 234605.56 | 234641.56 | 234789.17 |
| First-order autoregressive (df=18) | 234964.28 | 235000.28 | 235147.89 |

### DISCUSSION AND CONCLUSIONS

The present study demonstrates the application of IGC analyses using SPSS. We first describe the basic growth curve modeling framework and demonstrate how various growth curve models fit to empirical multiwave data via SPSS. To explore nonlinear changes with longitudinal time-structured data, we used IGC to examine the intra- and interindividual differences in longitudinal trajectories over time. Lastly, we examined the effectiveness of the program by comparing differences in the longitudinal patterns of positive youth development between the control and experimental groups. As the SPSS manuals on IGC do not have many examples in the intervention context, the present paper is a significant contribution to the literature. With reference to the lack of IGC studies in the social work literature, the present paper is a pioneer contribution to the field. Furthermore, it is noteworthy that very few longitudinal intervention studies have been conducted by employing this advanced technique in different Chinese contexts.

One of the strengths of IGC analyses is that the numbers of observations collected on each individual can maximize the degree to estimate a complex nonlinear growth curve model. The high number of time points allows us to model different types of polynomial growth curve models[51]. Furthermore, the power of the test is greatly improved by adding only a few additional waves of data collection[26,52]. Many researchers have commented on the need for conducting more developmental studies examining individual growth by using appropriate statistical methods, and using nonlinear growth curves to describe between- and within-individual change over time[1,9,10,16]. In particular, using a developmental approach in understanding the dynamic process of psychosocial development and risk-taking behaviors during adolescence may be useful for designing successful prevention programs[8,53,54,55]. Cleary, this study is a positive response and attempts to fill this research gap. We hope that this method will be more accessible to researchers in the field of social work and other allied professions.

### ACKNOWLEDGMENTS

The authorship of this paper is equally shared by the two authors. The preparation for this paper and the Project P.A.T.H.S. were financially supported by The Hong Kong Jockey Club Charities Trust.

## APPENDIX A

### Model 1: Unconditional Mean Model

**Model Dimension**

| | | Number of Levels | Covariance Structure | Number of Parameters | Subject Variables |
|---|---|---|---|---|---|
| Fixed Effects | Intercept | 1 | | 1 | |
| Random Effects | Intercept | 1 | Identity | 1 | id |
| Residual | | | | 1 | |
| Total | | 2 | | 3 | |

**Information Criteria**

| | |
|---|---|
| -2 Log Likelihood | 334969.033 |
| Akaike's Information Criterion (AIC) | 334975.033 |
| Hurvich and Tsai's Criterion (AICC) | 334975.034 |
| Bozdogan's Criterion (CAIC) | 335003.673 |
| Schwarz's Bayesian Criterion (BIC) | 335000.673 |

**Type III Tests of Fixed Effects**

| Source | Numerator df | Denominator df | F | Sig. |
|---|---|---|---|---|
| Intercept | 1 | 9631.527 | 449583.653 | 0.000 |

**Estimates of Fixed Effects**

| Parameter | Estimate | Std. Error | df | t | Sig. | 95% Confidence Interval | |
|---|---|---|---|---|---|---|---|
| | | | | | | Lower Bound | Upper Bound |
| Intercept | 155.581036 | 0.232034 | 9631.527 | 670.510 | 0.000 | 155.126201 | 156.035871 |

**Estimates of Covariance Parameters**

| Parameter | | Estimate | Std. Error | Wald Z | Sig. | 95% Confidence Interval | |
|---|---|---|---|---|---|---|---|
| | | | | | | Lower Bound | Upper Bound |
| Residual | | 228.744134 | 1.936866 | 118.100 | 0.000 | 224.979272 | 232.571997 |
| Intercept [subject = id] | Variance | 455.701266 | 7.712225 | 59.088 | 0.000 | 440.833529 | 471.070439 |

### Model 2: Unconditional Linear Growth Model

**Model Dimension**

| | | Number of Levels | Covariance Structure | Number of Parameters | Subject Variables |
|---|---|---|---|---|---|
| Fixed Effects | Intercept | 1 | | 1 | |
| | Time | 1 | | 1 | |
| Random Effects | Intercept + Time | 2 | Unstructured | 3 | id |
| Residual | | | | 1 | |
| Total | | 4 | | 6 | |

**Information Criteria**

| | |
|---|---|
| -2 Log Likelihood | 334025.838 |
| Akaike's Information Criterion (AIC) | 334037.838 |
| Hurvich and Tsai's Criterion (AICC) | 334037.841 |
| Bozdogan's Criterion (CAIC) | 334095.119 |
| Schwarz's Bayesian Criterion (BIC) | 334089.119 |

**Estimates of Fixed Effects**

| | | | | | | 95% Confidence Interval | |
|---|---|---|---|---|---|---|---|
| Parameter | Estimate | Std. Error | df | t | Sig. | Lower Bound | Upper Bound |
| Intercept | 155.910860 | 0.274810 | 8822.914 | 567.340 | 0.000 | 155.372167 | 156.449552 |
| Time | -0.328023 | 0.116614 | 6778.905 | -2.813 | 0.005 | -0.556623 | -0.099423 |

**Estimates of Covariance Parameters**

| | | | | | | 95% Confidence Interval | |
|---|---|---|---|---|---|---|---|
| Parameter | | Estimate | Std. Error | Wald Z | Sig. | Lower Bound | Upper Bound |
| Residual | | 192.113758 | 1.889325 | 101.684 | 0.000 | 188.446209 | 195.852685 |
| Intercept + Time [subject = id] | UN (1,1) | 532.888716 | 10.727355 | 49.676 | 0.000 | 512.272862 | 554.334232 |
| | UN (2,1) | -55.623597 | 3.644517 | -15.262 | 0.000 | -62.766718 | -48.480475 |
| | UN (2,2) | 42.537662 | 1.830458 | 23.239 | 0.000 | 39.097156 | 46.280929 |

### Model 3: Quadratic Growth Model

**Model Dimension**

| | | Number of Levels | Covariance Structure | Number of Parameters | Subject Variables |
|---|---|---|---|---|---|
| Fixed Effects | Intercept | 1 | | 1 | |
| | Time | 1 | | 1 | |
| | Time_sq | 1 | | 1 | |
| Random Effects | Intercept + Time | 2 | Unstructured | 3 | id |
| Residual | | | | 1 | |
| Total | | 5 | | 7 | |

**Information Criteria**

| | |
|---|---|
| -2 Log Likelihood | 333749.959 |
| Akaike's Information Criterion (AIC) | 333763.959 |
| Hurvich and Tsai's Criterion (AICC) | 333763.962 |
| Bozdogan's Criterion (CAIC) | 333830.786 |
| Schwarz's Bayesian Criterion (BIC) | 333823.786 |

**Estimates of Fixed Effects**

| Parameter | Estimate | Std. Error | df | t | Sig. | 95% Confidence Interval Lower Bound | 95% Confidence Interval Upper Bound |
|---|---|---|---|---|---|---|---|
| Intercept | 157.685999 | 0.294702 | 11121.113 | 535.069 | 0.000 | 157.108331 | 158.263667 |
| Time | -5.048185 | 0.306463 | 29742.869 | -16.472 | 0.000 | -5.648866 | -4.447505 |
| Time_sq | 1.747592 | 0.104963 | 25301.492 | 16.650 | 0.000 | 1.541859 | 1.953325 |

**Estimates of Covariance Parameters**

| Parameter | | Estimate | Std. Error | Wald Z | Sig. | 95% Confidence Interval Lower Bound | 95% Confidence Interval Upper Bound |
|---|---|---|---|---|---|---|---|
| Residual | | 190.085974 | 1.867786 | 101.771 | 0.000 | 186.460206 | 193.782246 |
| Intercept + Time [subject = id] | UN (1,1) | 533.975708 | 10.706157 | 49.876 | 0.000 | 513.398977 | 555.377142 |
| | UN (2,1) | -55.958842 | 3.626588 | -15.430 | 0.000 | -63.066823 | -48.850860 |
| | UN (2,2) | 42.535485 | 1.815904 | 23.424 | 0.000 | 39.121213 | 46.247735 |

### Model 4: Cubic Growth Model

**Model Dimension**

| | | Number of Levels | Covariance Structure | Number of Parameters | Subject Variables |
|---|---|---|---|---|---|
| Fixed Effects | Intercept | 1 | | 1 | |
| | Time | 1 | | 1 | |
| | Time_sq | 1 | | 1 | |
| | Time_cub | 1 | | 1 | |
| Random Effects | Intercept + Time | 2 | Unstructured | 3 | id |
| Residual | | | | 1 | |
| Total | | 6 | | 8 | |

**Information Criteria**

| | |
|---|---|
| -2 Log Likelihood | 333684.554 |
| Akaike's Information Criterion (AIC) | 333700.554 |
| Hurvich and Tsai's Criterion (AICC) | 333700.558 |
| Bozdogan's Criterion (CAIC) | 333776.928 |
| Schwarz's Bayesian Criterion (BIC) | 333768.928 |

**Estimates of Fixed Effects**

| | | | | | | 95% Confidence Interval | |
|---|---|---|---|---|---|---|---|
| Parameter | Estimate | Std. Error | df | t | Sig. | Lower Bound | Upper Bound |
| Intercept | 158.200143 | 0.301451 | 12002.213 | 524.796 | 0.000 | 157.609251 | 158.791035 |
| Time | -9.459394 | 0.625204 | 25657.553 | -15.130 | 0.000 | -10.684829 | -8.233958 |
| Time_sq | 6.261327 | 0.567495 | 23457.836 | 11.033 | 0.000 | 5.148999 | 7.373655 |
| Time_cub | -1.122656 | 0.138710 | 23043.425 | -8.094 | 0.000 | -1.394538 | -0.850774 |

**Estimates of Covariance Parameters**

| | | | | | | 95% Confidence Interval | |
|---|---|---|---|---|---|---|---|
| Parameter | | Estimate | Std. Error | Wald Z | Sig. | Lower Bound | Upper Bound |
| Residual | | 189.500331 | 1.862289 | 101.757 | 0.000 | 185.885238 | 193.185729 |
| Intercept + Time [subject = id] | UN (1,1) | 534.401214 | 10.704881 | 49.921 | 0.000 | 513.826567 | 555.799710 |
| | UN (2,1) | -56.258192 | 3.627004 | -15.511 | 0.000 | -63.366989 | -49.149395 |
| | UN (2,2) | 42.749224 | 1.817371 | 23.523 | 0.000 | 39.331602 | 46.463812 |

### Model 5: A Controlled Model with Predictors

**Model Dimension**

| | | Number of Levels | Covariance Structure | Number of Parameters | Subject Variables |
|---|---|---|---|---|---|
| Fixed Effects | Intercept | 1 | | 1 | |
| | Time | 1 | | 1 | |
| | Time_sq | 1 | | 1 | |
| | Time_cub | 1 | | 1 | |
| | group | 1 | | 1 | |
| | k2 | 1 | | 1 | |
| | age | 1 | | 1 | |
| | group * Time | 1 | | 1 | |
| | group * Time_sq | 1 | | 1 | |
| | group * Time_cub | 1 | | 1 | |
| | k2 * Time | 1 | | 1 | |
| | k2 * Time_sq | 1 | | 1 | |
| | k2 * Time_cub | 1 | | 1 | |
| | age * Time | 1 | | 1 | |
| | age * Time_sq | 1 | | 1 | |
| | age * Time_cub | 1 | | 1 | |
| Random Effects | Intercept + Time | 2 | Unstructured | 3 | id |
| Residual | | | | 1 | |
| Total | | 18 | | 20 | |

**Information Criteria**

| | |
|---|---|
| -2 Log Likelihood | 233789.704 |
| Akaike's Information Criterion (AIC) | 233829.704 |
| Hurvich and Tsai's Criterion (AICC) | 233829.735 |
| Bozdogan's Criterion (CAIC) | 234013.718 |
| Schwarz's Bayesian Criterion (BIC) | 233993.718 |

**Estimates of Fixed Effects**

| Parameter | Estimate | Std. Error | df | t | Sig. | 95% Confidence Interval | |
|---|---|---|---|---|---|---|---|
| | | | | | | Lower Bound | Upper Bound |
| Intercept | 156.092067 | 0.499113 | 7690.645 | 312.739 | 0.000 | 155.113669 | 157.070465 |
| Time | -5.622431 | 0.997006 | 18597.867 | -5.639 | 0.000 | -7.576654 | -3.668209 |
| Time_sq | 4.076358 | 0.920552 | 17140.516 | 4.428 | 0.000 | 2.271981 | 5.880735 |
| Time_cub | -0.760958 | 0.227309 | 16900.036 | -3.348 | 0.001 | -1.206507 | -0.315409 |
| group | 0.057759 | 0.357038 | 7489.439 | 0.162 | 0.871 | -0.642136 | 0.757654 |
| k2 | 5.961435 | 0.703101 | 7462.870 | 8.479 | 0.000 | 4.583158 | 7.339711 |
| age | -1.686331 | 0.391491 | 7413.377 | -4.307 | 0.000 | -2.453764 | -0.918898 |
| group * Time | 2.921715 | 0.701601 | 18243.057 | 4.164 | 0.000 | 1.546512 | 4.296918 |
| group * Time_sq | -2.381432 | 0.652016 | 16943.156 | -3.652 | 0.000 | -3.659452 | -1.103412 |
| group * Time_cub | 0.534979 | 0.161594 | 16751.241 | 3.311 | 0.001 | 0.218238 | 0.851721 |
| k2 * Time | -9.876641 | 1.380947 | 18282.678 | -7.152 | 0.000 | -12.583428 | -7.169855 |
| k2 * Time_sq | 6.117306 | 1.278788 | 16907.613 | 4.784 | 0.000 | 3.610749 | 8.623864 |
| k2 * Time_cub | -1.156036 | 0.316100 | 16727.092 | -3.657 | 0.000 | -1.775624 | -0.536447 |
| age * Time | 3.191449 | 0.805365 | 18480.635 | 3.963 | 0.000 | 1.612860 | 4.770038 |
| age * Time_sq | -2.199849 | 0.755895 | 17145.136 | -2.910 | 0.004 | -3.681479 | -0.718218 |
| age * Time_cub | 0.449315 | 0.187762 | 16915.750 | 2.393 | 0.017 | 0.081281 | 0.817348 |

**Estimates of Covariance Parameters**

| Parameter | | Estimate | Std. Error | Wald Z | Sig. | 95% Confidence Interval | |
|---|---|---|---|---|---|---|---|
| | | | | | | Lower Bound | Upper Bound |
| Residual | | 179.357492 | 2.026818 | 88.492 | 0.000 | 175.428671 | 183.374300 |
| Intercept + Time [subject = id] | UN (1,1) | 502.629872 | 11.869756 | 42.345 | 0.000 | 479.895762 | 526.440966 |
| | UN (2,1) | -48.229541 | 3.859513 | -12.496 | 0.000 | -55.794049 | -40.665034 |
| | UN (2,2) | 41.665972 | 1.952382 | 21.341 | 0.000 | 38.009834 | 45.673792 |

### Model 6: Testing Error Structure Covariance Structure (Unstructured)

**Model Dimension**

| | | Number of Levels | Covariance Structure | Number of Parameters | Subject Variables | Number of Subjects |
|---|---|---|---|---|---|---|
| Fixed Effects | Intercept | 1 | | 1 | | |
| | Time | 1 | | 1 | | |
| | Time_sq | 1 | | 1 | | |
| | Time_cub | 1 | | 1 | | |
| | group | 1 | | 1 | | |
| | k2 | 1 | | 1 | | |
| | age | 1 | | 1 | | |
| | group * Time | 1 | | 1 | | |
| | group * Time_sq | 1 | | 1 | | |
| | group * Time_cub | 1 | | 1 | | |
| | k2 * Time | 1 | | 1 | | |
| | k2 * Time_sq | 1 | | 1 | | |
| | k2 * Time_cub | 1 | | 1 | | |
| | age * Time | 1 | | 1 | | |
| | age * Time_sq | 1 | | 1 | | |
| | age * Time_cub | 1 | | 1 | | |
| Repeated Effects | Wave | 6 | Unstructured | 21 | id | 5989 |
| Total | | 22 | | 37 | | |

**Information Criteria**

| | |
|---|---|
| -2 Log Likelihood | 233203.108 |
| Akaike's Information Criterion (AIC) | 233277.108 |
| Hurvich and Tsai's Criterion (AICC) | 233277.213 |
| Bozdogan's Criterion (CAIC) | 233617.534 |
| Schwarz's Bayesian Criterion (BIC) | 233580.534 |

**Type III Tests of Fixed Effects**

| Source | Numerator df | Denominator df | F | Sig. |
|---|---|---|---|---|
| Intercept | 1 | 5627.635 | 103715.759 | 0.000 |
| Time | 1 | 5004.428 | 29.901 | 0.000 |
| Time_sq | 1 | 4897.586 | 18.227 | 0.000 |
| Time_cub | 1 | 4794.049 | 10.350 | 0.001 |
| group | 1 | 5531.181 | .213 | 0.645 |
| k2 | 1 | 5515.433 | 70.275 | 0.000 |
| age | 1 | 5502.432 | 19.225 | 0.000 |
| group * Time | 1 | 4817.472 | 14.142 | 0.000 |
| group * Time_sq | 1 | 4734.701 | 11.842 | 0.001 |
| group * Time_cub | 1 | 4649.853 | 10.553 | 0.001 |
| k2 * Time | 1 | 4772.038 | 40.269 | 0.000 |
| k2 * Time_sq | 1 | 4664.874 | 18.620 | 0.000 |
| k2 * Time_cub | 1 | 4587.926 | 11.336 | 0.001 |
| age * Time | 1 | 4981.409 | 12.345 | 0.000 |
| age * Time_sq | 1 | 4897.211 | 6.720 | 0.010 |
| age * Time_cub | 1 | 4826.110 | 4.678 | 0.031 |

**Estimates of Fixed Effects**

| Parameter | Estimate | Std. Error | df | t | Sig. | 95% Confidence Interval | |
|---|---|---|---|---|---|---|---|
| | | | | | | Lower Bound | Upper Bound |
| Intercept | 156.197981 | 0.485013 | 5627.635 | 322.049 | 0.000 | 155.247169 | 157.148793 |
| Time | -5.891237 | 1.077373 | 5004.428 | -5.468 | 0.000 | -8.003360 | -3.779113 |
| Time_sq | 4.170288 | 0.976812 | 4897.586 | 4.269 | 0.000 | 2.255299 | 6.085278 |
| Time_cub | -0.760124 | 0.236270 | 4794.049 | -3.217 | 0.001 | -1.223322 | -0.296925 |
| group | 0.159797 | 0.346380 | 5531.181 | 0.461 | 0.645 | -0.519243 | 0.838838 |
| k2 | 5.716551 | 0.681921 | 5515.433 | 8.383 | 0.000 | 4.379716 | 7.053385 |
| age | -1.664635 | 0.379656 | 5502.432 | -4.385 | 0.000 | -2.408910 | -0.920359 |
| group * Time | 2.855923 | 0.759435 | 4817.472 | 3.761 | 0.000 | 1.367084 | 4.344763 |
| group * Time_sq | -2.385236 | 0.693143 | 4734.701 | -3.441 | 0.001 | -3.744118 | -1.026353 |
| group * Time_cub | 0.546584 | 0.168257 | 4649.853 | 3.249 | 0.001 | 0.216721 | 0.876447 |
| k2 * Time | -9.487996 | 1.495173 | 4772.038 | -6.346 | 0.000 | -12.419226 | -6.556767 |
| k2 * Time_sq | 5.866834 | 1.359605 | 4664.874 | 4.315 | 0.000 | 3.201366 | 8.532303 |
| k2 * Time_cub | -1.108123 | 0.329117 | 4587.926 | -3.367 | 0.001 | -1.753351 | -0.462895 |
| age * Time | 3.062785 | 0.871694 | 4981.409 | 3.514 | 0.000 | 1.353881 | 4.771689 |
| age * Time_sq | -2.080538 | 0.802587 | 4897.211 | -2.592 | 0.010 | -3.653969 | -0.507107 |
| age * Time_cub | 0.422278 | 0.195241 | 4826.110 | 2.163 | 0.031 | 0.039517 | 0.805039 |

**Estimates of Covariance Parameters**

| Parameter | | Estimate | Std. Error | Wald Z | Sig. | 95% Confidence Interval | |
|---|---|---|---|---|---|---|---|
| | | | | | | Lower Bound | Upper Bound |
| Repeated Measures | UN (1,1) | 638.407599 | 12.639803 | 50.508 | 0.000 | 614.108556 | 663.668107 |
| | UN (2,1) | 426.909562 | 10.919939 | 39.095 | 0.000 | 405.506875 | 448.312249 |
| | UN (2,2) | 662.060866 | 13.038897 | 50.776 | 0.000 | 636.992043 | 688.116274 |
| | UN (3,1) | 438.289142 | 11.370749 | 38.545 | 0.000 | 416.002884 | 460.575399 |
| | UN (3,2) | 522.003376 | 11.955621 | 43.662 | 0.000 | 498.570790 | 545.435962 |
| | UN (3,3) | 693.681400 | 14.191581 | 48.880 | 0.000 | 666.416692 | 722.061574 |
| | UN (4,1) | 377.632358 | 10.848532 | 34.810 | 0.000 | 356.369626 | 398.895090 |
| | UN (4,2) | 440.197292 | 11.267176 | 39.069 | 0.000 | 418.114032 | 462.280552 |
| | UN (4,3) | 487.355792 | 11.982242 | 40.673 | 0.000 | 463.871029 | 510.840555 |
| | UN (4,4) | 646.323091 | 13.532161 | 47.762 | 0.000 | 620.337365 | 673.397351 |
| | UN (5,1) | 369.754865 | 10.991681 | 33.640 | 0.000 | 348.211566 | 391.298164 |
| | UN (5,2) | 431.534013 | 11.470414 | 37.621 | 0.000 | 409.052415 | 454.015611 |
| | UN (5,3) | 482.754707 | 12.121497 | 39.826 | 0.000 | 458.997010 | 506.512405 |
| | UN (5,4) | 517.057269 | 12.236376 | 42.256 | 0.000 | 493.074412 | 541.040126 |
| | UN (5,5) | 671.913564 | 14.064528 | 47.774 | 0.000 | 644.905403 | 700.052806 |
| | UN (6,1) | 338.006363 | 10.711142 | 31.557 | 0.000 | 317.012910 | 358.999817 |
| | UN (6,2) | 383.998577 | 11.012258 | 34.870 | 0.000 | 362.414948 | 405.582205 |
| | UN (6,3) | 435.359380 | 11.729875 | 37.115 | 0.000 | 412.369247 | 458.349513 |
| | UN (6,4) | 451.118358 | 11.596361 | 38.902 | 0.000 | 428.389909 | 473.846808 |
| | UN (6,5) | 498.010846 | 12.196057 | 40.834 | 0.000 | 474.107015 | 521.914678 |
| | UN (6,6) | 637.377586 | 13.647840 | 46.702 | 0.000 | 611.181843 | 664.696100 |

### Model 7: Testing Error Structure Covariance Structure (Compound Symmetry)

**Model Dimension**

| | | Number of Levels | Covariance Structure | Number of Parameters | Subject Variables | Number of Subjects |
|---|---|---|---|---|---|---|
| Fixed Effects | Intercept | 1 | | 1 | | |
| | Time | 1 | | 1 | | |
| | Time_sq | 1 | | 1 | | |
| | Time_cub | 1 | | 1 | | |
| | group | 1 | | 1 | | |
| | k2 | 1 | | 1 | | |
| | age | 1 | | 1 | | |
| | group * Time | 1 | | 1 | | |
| | group * Time_sq | 1 | | 1 | | |
| | group * Time_cub | 1 | | 1 | | |
| | k2 * Time | 1 | | 1 | | |
| | k2 * Time_sq | 1 | | 1 | | |
| | k2 * Time_cub | 1 | | 1 | | |
| | age * Time | 1 | | 1 | | |
| | age * Time_sq | 1 | | 1 | | |
| | age * Time_cub | 1 | | 1 | | |
| Repeated Effects | Wave | 6 | Compound Symmetry | 2 | id | 5989 |
| Total | | 22 | | 18 | | |

**Information Criteria**

| | |
|---|---|
| -2 Log Likelihood | 234605.557 |
| Akaike's Information Criterion (AIC) | 234641.557 |
| Hurvich and Tsai's Criterion (AICC) | 234641.583 |
| Bozdogan's Criterion (CAIC) | 234807.170 |
| Schwarz's Bayesian Criterion (BIC) | 234789.170 |

**Type III Tests of Fixed Effects**

| Source | Numerator df | Denominator df | F | Sig. |
|---|---|---|---|---|
| Intercept | 1 | 11574.166 | 101885.761 | 0.000 |
| Time | 1 | 21502.043 | 28.881 | 0.000 |
| Time_sq | 1 | 21335.190 | 16.878 | 0.000 |
| Time_cub | 1 | 21270.148 | 9.333 | 0.002 |
| group | 1 | 11238.794 | 0.074 | 0.785 |
| k2 | 1 | 11198.869 | 74.278 | 0.000 |
| age | 1 | 11142.523 | 20.103 | 0.000 |
| group * Time | 1 | 21345.056 | 14.935 | 0.000 |
| group * Time_sq | 1 | 21251.676 | 11.698 | 0.001 |
| group * Time_cub | 1 | 21211.234 | 9.627 | 0.002 |
| k2 * Time | 1 | 21381.707 | 43.545 | 0.000 |
| k2 * Time_sq | 1 | 21259.477 | 19.046 | 0.000 |
| k2 * Time_cub | 1 | 21209.862 | 11.129 | 0.001 |
| age * Time | 1 | 21755.205 | 15.271 | 0.000 |
| age * Time_sq | 1 | 21483.894 | 8.340 | 0.004 |
| age * Time_cub | 1 | 21377.819 | 5.553 | 0.018 |

**Estimates of Fixed Effects**

| | | | | | | 95% Confidence Interval | |
|---|---|---|---|---|---|---|---|
| Parameter | Estimate | Std. Error | df | t | Sig. | Lower Bound | Upper Bound |
| Intercept | 156.183077 | 0.489302 | 11574.166 | 319.195 | 0.000 | 155.223962 | 157.142192 |
| Time | -5.750023 | 1.069950 | 21502.043 | -5.374 | 0.000 | -7.847204 | -3.652841 |
| Time_sq | 4.104453 | 0.999070 | 21335.190 | 4.108 | 0.000 | 2.146201 | 6.062705 |
| Time_cub | -0.754965 | 0.247125 | 21270.148 | -3.055 | 0.002 | -1.239348 | -0.270581 |
| group | 0.095390 | 0.350253 | 11238.794 | 0.272 | 0.785 | -0.591166 | 0.781947 |
| k2 | 5.943140 | 0.689583 | 11198.869 | 8.618 | 0.000 | 4.591436 | 7.294843 |
| age | -1.721759 | 0.384011 | 11142.523 | -4.484 | 0.000 | -2.474489 | -0.969030 |
| group * Time | 2.918109 | 0.755093 | 21345.056 | 3.865 | 0.000 | 1.438070 | 4.398149 |
| group * Time_sq | -2.424246 | 0.708797 | 21251.676 | -3.420 | 0.001 | -3.813542 | -1.034950 |
| group * Time_cub | 0.545770 | 0.175903 | 21211.234 | 3.103 | 0.002 | 0.200986 | 0.890554 |
| k2 * Time | -9.805659 | 1.485965 | 21381.707 | -6.599 | 0.000 | -12.718262 | -6.893056 |
| k2 * Time_sq | 6.067977 | 1.390410 | 21259.477 | 4.364 | 0.000 | 3.342667 | 8.793287 |
| k2 * Time_cub | -1.148001 | 0.344120 | 21209.862 | -3.336 | 0.001 | -1.822503 | -0.473499 |
| age * Time | 3.387648 | 0.866881 | 21755.205 | 3.908 | 0.000 | 1.688499 | 5.086798 |
| age * Time_sq | -2.370938 | 0.820985 | 21483.894 | -2.888 | 0.004 | -3.980129 | -0.761747 |
| age * Time_cub | 0.481194 | 0.204205 | 21377.819 | 2.356 | 0.018 | 0.080938 | 0.881451 |

**Estimates of Covariance Parameters**

| | | | | | | 95% Confidence Interval | |
|---|---|---|---|---|---|---|---|
| Parameter | | Estimate | Std. Error | Wald Z | Sig. | Lower Bound | Upper Bound |
| Repeated Measures | CS diagonal offset | 216.432846 | 2.117975 | 102.189 | 0.000 | 212.321246 | 220.624067 |
| | CS covariance | 441.579372 | 9.213111 | 47.929 | 0.000 | 423.522006 | 459.636739 |

### Model 8: Testing Error Structure Covariance Structure (First-Order Autoregressive)

**Model Dimension**

| | | Number of Levels | Covariance Structure | Number of Parameters | Subject Variables | Number of Subjects |
|---|---|---|---|---|---|---|
| Fixed Effects | Intercept | 1 | | 1 | | |
| | Time | 1 | | 1 | | |
| | Time_sq | 1 | | 1 | | |
| | Time_cub | 1 | | 1 | | |
| | group | 1 | | 1 | | |
| | k2 | 1 | | 1 | | |
| | age | 1 | | 1 | | |
| | group * Time | 1 | | 1 | | |
| | group * Time_sq | 1 | | 1 | | |
| | group *Time_cub | 1 | | 1 | | |
| | k2 * Time | 1 | | 1 | | |
| | k2 * Time_sq | 1 | | 1 | | |
| | k2 * Time_cub | 1 | | 1 | | |
| | age * Time | 1 | | 1 | | |
| | age * Time_sq | 1 | | 1 | | |
| | age * Time_cub | 1 | | 1 | | |
| Repeated Effects | Wave | 6 | First-Order Autoregressive | 2 | id | 5989 |
| Total | | 22 | | 18 | | |

**Information Criteria**

| | |
|---|---|
| -2 Log Likelihood | 234964.276 |
| Akaike's Information Criterion (AIC) | 235000.276 |
| Hurvich and Tsai's Criterion (AICC) | 235000.301 |
| Bozdogan's Criterion (CAIC) | 235165.888 |
| Schwarz's Bayesian Criterion (BIC) | 235147.888 |

**Type III Tests of Fixed Effects**

| Source | Numerator df | Denominator df | F | Sig. |
|---|---|---|---|---|
| Intercept | 1 | 14292.987 | 101122.057 | 0.000 |
| Time | 1 | 19629.239 | 34.445 | 0.000 |
| Time_sq | 1 | 19307.449 | 18.495 | 0.000 |
| Time_cub | 1 | 19083.981 | 9.556 | 0.002 |
| group | 1 | 13924.929 | 0.132 | 0.716 |
| k2 | 1 | 13886.084 | 80.720 | 0.000 |
| age | 1 | 13777.756 | 20.502 | 0.000 |
| group * Time | 1 | 19387.241 | 18.390 | 0.000 |
| group * Time_sq | 1 | 19313.616 | 11.895 | 0.001 |
| group * Time_cub | 1 | 19121.082 | 9.970 | 0.002 |
| k2 * Time | 1 | 19403.874 | 52.730 | 0.000 |
| k2 * Time_sq | 1 | 19181.676 | 21.738 | 0.000 |
| k2 * Time_cub | 1 | 18989.049 | 12.360 | 0.000 |
| age * Time | 1 | 19633.129 | 13.368 | 0.000 |
| age * Time_sq | 1 | 19344.736 | 5.211 | 0.022 |
| age * Time_cub | 1 | 19122.967 | 3.060 | 0.080 |

**Estimates of Fixed Effects**

| Parameter | Estimate | Std. Error | df | t | Sig. | 95% Confidence Interval | |
|---|---|---|---|---|---|---|---|
| | | | | | | Lower Bound | Upper Bound |
| Intercept | 156.260684 | 0.491391 | 14292.987 | 317.997 | 0.000 | 155.297495 | 157.223874 |
| Time | -6.010577 | 1.024131 | 19629.239 | -5.869 | 0.000 | -8.017960 | -4.003195 |
| Time_sq | 4.169871 | 0.969606 | 19307.449 | 4.301 | 0.000 | 2.269360 | 6.070383 |
| Time_cub | -0.737307 | 0.238513 | 19083.981 | -3.091 | 0.002 | -1.204815 | -0.269800 |
| group | -0.127482 | 0.350730 | 13924.929 | -0.363 | 0.716 | -0.814960 | 0.559996 |
| k2 | 6.206854 | 0.690847 | 13886.084 | 8.984 | 0.000 | 4.852702 | 7.561007 |
| age | -1.742834 | 0.384904 | 13777.756 | -4.528 | 0.000 | -2.497299 | -0.988369 |
| group * Time | 3.082291 | 0.718751 | 19387.241 | 4.288 | 0.000 | 1.673477 | 4.491104 |
| group * Time_sq | -2.372401 | 0.687858 | 19313.616 | -3.449 | 0.001 | -3.720662 | -1.024140 |
| group * Time_cub | 0.536890 | 0.170034 | 19121.082 | 3.158 | 0.002 | 0.203608 | 0.870171 |
| k2 * Time | -10.301498 | 1.418634 | 19403.874 | -7.262 | 0.000 | -13.082144 | -7.520853 |
| k2 * Time_sq | 6.287249 | 1.348485 | 19181.676 | 4.662 | 0.000 | 3.644101 | 8.930398 |
| k2 * Time_cub | -1.167575 | 0.332101 | 18989.049 | -3.516 | 0.000 | -1.818522 | -0.516627 |
| age * Time | 3.031664 | 0.829177 | 19633.129 | 3.656 | 0.000 | 1.406407 | 4.656920 |
| age * Time_sq | -1.819882 | 0.797233 | 19344.736 | -2.283 | 0.022 | -3.382527 | -0.257236 |
| age * Time_cub | 0.344826 | 0.197109 | 19122.967 | 1.749 | 0.080 | -0.041525 | 0.731177 |

**Estimates of Covariance Parameters**

| Parameter | | Estimate | Std. Error | Wald Z | Sig. | 95% Confidence Interval | |
|---|---|---|---|---|---|---|---|
| | | | | | | Lower Bound | Upper Bound |
| Repeated Measures | AR1 diagonal | 655.667926 | 8.323071 | 78.777 | 0.000 | 639.556266 | 672.185470 |
| | AR1 rho | 0.746156 | 0.003678 | 202.862 | 0.000 | 0.738859 | 0.753278 |
